# Stabilized designs of the malaria adhesin protein PvRBP2b for use as a potential diagnostic for *Plasmodium vivax*

**DOI:** 10.1016/j.jbc.2025.108290

**Published:** 2025-02-10

**Authors:** Jaison D Sa, Lucas Krauss, Lauren Smith, Laura D'Andrea, Li-Jin Chan, Anju Abraham, Nicholas Kiernan-Walker, Ramin Mazhari, Macie Lamont, Pailene S. Lim, Jetsumon Sattabongkot, Marcus VG. Lacerda, Lyndes Wini, Ivo Mueller, Rhea J. Longley, Phillip Pymm, Sarel J. Fleishman, Wai-Hong Tham

**Affiliations:** 1 The Walter and Eliza Hall Institute of Medical Research, Parkville, Victoria, Australia; 2 Department of Medical Biology, The University of Melbourne, Melbourne, Victoria, Australia; 3 Department of Biomolecular Sciences, Weizmann Institute of Science, Rehovot, Israel; 4 Mahidol Vivax Research Unit, Faculty of Tropical Medicine, Mahidol University, Bangkok, Thailand; 5 Fundação de Medicina Tropical Dr Heitor Vieira Dourado and Instituto de Pesquisa Leônidas e Maria Deane, Fiocruz, Manaus, Brazil; 6 National Vector Borne Disease Control Programme, Ministry of Health and Medical Services, Honiara, The Solomon Islands; 7 Research School of Biology, The Australian National University, Canberra, ACT, Australia

**Keywords:** malaria, PROSS, antigen design, protein stability, serology

## Abstract

*Plasmodium vivax* is emerging as the most prevalent species causing malaria outside Africa. Most *P. vivax* infections are relapses due to the reactivation of the dormant liver stage parasites (hypnozoites). Hypnozoites are a major reservoir for transmission but undetectable by commercial diagnostic tests. Antibodies against *P. vivax* reticulocyte-binding protein 2b (PvRBP2b) are among the most reliable serological biomarkers for recent *P. vivax* infections in the prior 9 months and act as indirect biomarkers for risk of relapse. We sought to design stabilized variants of PvRBP2b, under stringent conditions of minimally perturbing the solvent-accessible surfaces to maintain its antigenicity profile. Furthermore, for some of the designs, due to limited diversity of natural PvRBP2b homologs, we combined AI-based ProteinMPNN and PROSS atomistic design calculations. The best, bearing 19 core mutations relative to PvRBP2b, expressed 16-fold greater amounts (up to 11 mg/l), and had 14 °C higher thermal tolerance than the parental protein. Critically, the stabilized designs retained binding to naturally acquired human mAbs with nanomolar affinities, suggesting that the immunologically competent surfaces were retained as was confirmed by crystallographic analyses. Using longitudinal observational cohorts from malaria endemic regions of Thailand, Brazil, and the Solomon Islands, we show that antibody responses against the designs are highly correlated with those against the parental protein and can classify individuals as recently infected with *P. vivax.* This efficient computational stability design methodology can be used to enhance the biophysical properties of other recalcitrant proteins for use as diagnostics or vaccine immunogens.

*Plasmodium vivax* remains a key obstacle to malaria elimination. *P. vivax* is the most widely distributed parasite with estimated 4 to 7 million annual cases in Asia, Oceania, and the Americas (1). A major challenge to *P. vivax* elimination is its dormant form, the hypnozoite, which evades existing control interventions to cause relapsing infections (2, 3). By doing so, the parasite can maintain “hidden” low-density infections that sustain ongoing transmission.

Due to dormancy, individuals carrying hypnozoites are clinically silent yet are a reservoir for transmission (4, 5). A recent in-development diagnostic is currently able to detect *P. vivax* infections within the prior 9 months with 80% sensitivity and 80% specificity (5). This diagnostic comprises a panel of eight *P. vivax* proteins that serve as serological exposure markers capable of classifying individuals with recent *P. vivax* infections who have a high likelihood of harboring hypnozoites and should be targeted with antihypnozoite therapy (6). Among these markers, the *P. vivax* reticulocyte-binding protein 2b (PvRBP2b), a member of the PvRBP family of protein adhesins (7, 8), produces the top prediction for dormant *P. vivax* infection (5). PvRBP2b binds to human transferrin receptor 1 (TfR1) to mediate entry into reticulocytes (9, 10). In addition, it is a target of naturally acquired immunity (11, 12, 13, 14, 15, 16, 17, 18, 19), and several longitudinal cohort studies show that antibodies to PvRBP2b are correlated with clinical protection (20, 21). Naturally acquired human mAbs against PvRBP2b functionally inhibit its binding to reticulocytes and block the interaction between PvRBP2b and TfR1 (22).

PvRBP2b can be produced in recombinant microbial systems resulting in a well-folded and functional protein (9, 10). Nevertheless, its expression yields and stability are low, limiting its development as a serological marker for diagnostic tests, particularly for use public health interventions such as serological testing and treatment in endemic countries, which are targeting malaria elimination (23). For example, recently released World Health Organization preferred product characteristics for a diagnostic test for risk of *P. vivax* relapse, call for a test shelf-life of ≥18 months at ≥35 °C and 90% relative humidity (4). Several computational and experimental approaches have been developed to address marginal protein stability and low expressibility. Typically, stabilizing mutations are introduced in solvent-exposed positions because those are less likely to compromise protein foldability (24). In the case of PvRBP2b, however, structural analyses indicated that >48% of the solvent-accessible surface is targeted by naturally acquired human antibodies from previously infected individuals (22). To serve as a useful serological marker, the designed antigen must exhibit nearly the same antigenic profile as parental PvRBP2b, dictating that much of the surface should be free of mutations, and that the natural protein backbone conformation must be carefully maintained. Therefore, the design strategy should be able to introduce core mutations without, however, perturbing the backbone structure.

Introducing many stabilizing core mutations while mostly maintaining the protein surface demands very high accuracy in design. Due to these stringent requirements, we applied the computational stability-design algorithm PROSS (25). PROSS has been successfully applied to dozens of different enzymes (26), binders (27), and vaccine immunogens (28), including *Plasmodium falciparum* reticulocyte-binding homolog 5 (PfRh5), the leading vaccine candidate against *P. falciparum* blood stages (29, 30, 31). In many cases, including PfRh5, designs exhibited much improved thermal and kinetic stability and expressibility without impacting protein activity, and the stabilized PfRh5 design has recently entered phase II clinical trials in West Africa (32). Although the previous successful applications of PROSS are encouraging, to the best of our knowledge, this approach has not been applied to design diagnostic antigens in which surface mutations were strongly limited. A further complication is that PROSS relies on a multiple-sequence alignment (MSA) of homologs to restrict atomistic design choices to amino acid identities that are commonly observed in the natural diversity. This aspect of the PROSS methodology is critical to its ability to design dozens of simultaneous mutations in large and complex proteins without leading to aggregation, misfolding, and loss of function (33, 34). Due to the limited natural diversity of *Plasmodia* species, however, we only detected a few dozen PvRBP2b homologs in sequence databases. We therefore combined the recent structure- and AI-based ProteinMPNN approach with the atomistic design calculations of PROSS, thus circumventing the requirement for a deep MSA.

Using this combined workflow, we generated stabilized PvRBP2b designs with their immunogenic properties retained. The best of the nine experimentally tested PROSS designs exhibited higher yields and increased thermal stability while maintaining binding to all naturally acquired human mAbs in our panel. Using a multiplex approach, our results show that the PvRBP2b designs maintained the same sensitivity and specificity for classifying recent *P. vivax* infection as the parental protein in serological tests. In addition to providing a promising candidate for an urgently needed diagnostic kit for malaria elimination, our study provides a blueprint for future efforts to stabilize antigens for use as diagnostics and vaccine immunogens.

## Results

### Computational design

**Figure 1:**
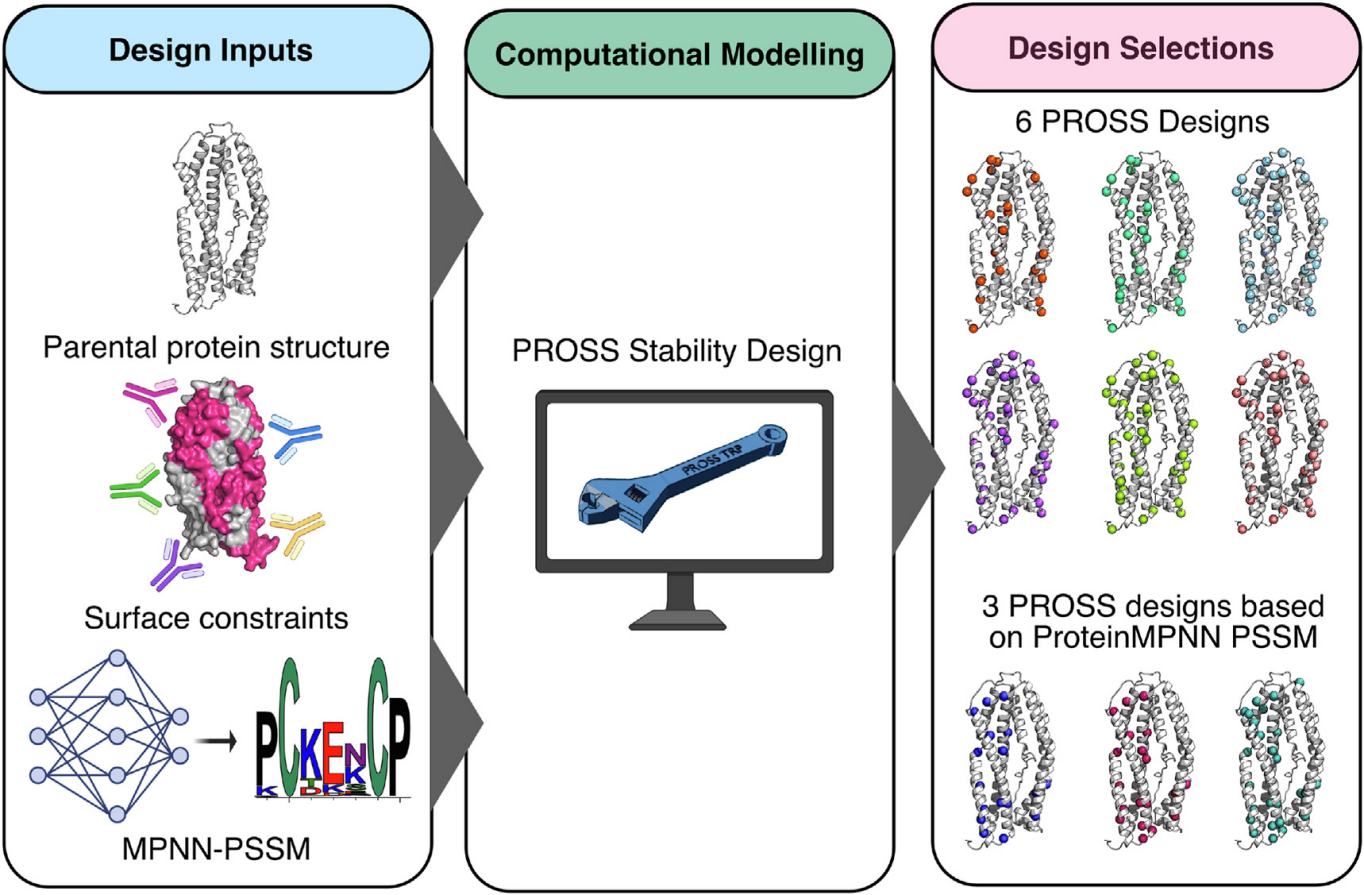
**Design process of stabilized PvRBP2b**_**169-470**_**variants.** Stabilized designs of PvRBP2b were computed under stringent conditions of minimally perturbing the solvent-accessible surfaces to maintain its antigenicity profile. Due to limited diversity of natural PvRBP2b homologs, in some designs, we used AI-based ProteinMPNN to generate a pseudo-PSSM followed by PROSS atomistic design calculations. A total of nine designs were tested. PvRBP2b, *Plasmodium vivax* reticulocyte-binding protein 2b.

PvRBP2b is a 326 kDa protein with a putative red blood cell binding domain and a C-terminal transmembrane region. The surface of the N-terminal domain of the natural protein PvRBP2b (residues 169–470, PvRBP2b_169-470_) is a mostly positively charged α-helical protein comprising ten α-helices and two very short antiparallel β-sheets (9). The crystal structure of PvRBP2b_169-470_ has two disulfide bonds (9, 22). All natural fragments with the N-terminal domain bound reticulocytes (PvRBP2b_161-1454_, PvRBP2b_161-969_, PvRBP2b_169-813_, and PvRBP2b_169-652_), whereas their corresponding fragments without the domain did not (PvRBP2b_474-1454_ and PvRBP2b_474-969_). We have previously shown that PvRBP2b_169-470_ can achieve equal sensitivity and specificity at predicting recent *P. vivax* exposure compared to its larger counterpart PvRBP2b_161-1454_ (15). Therefore, we designed stabilized variants using PvRBP2b_169-470_ as the template sequence and will refer to this sequence as the parental construct (Fig. 1).

The conventional PROSS stability design algorithm starts by searching the nonredundant sequence database for homologs and aligning them (25, 35). The resulting MSA is then used to generate a statistical model of substitutions at each amino acid position (position-specific scoring matrix; PSSM) to guide Rosetta atomistic design choices (36). The atomistic design step uses a physics-based energy function (37), dominated by van der Waals interactions, electrostatics, hydrogen bonding, and solvation, to optimize the native-state stability. During design calculations, mutations that are rare according to the PSSM are disallowed, and the remaining mutations are weighted according to their occurrence. Like many surface antigens from *Plasmodia*, however, PvRBP2b exhibits significant homology to only a few dozen sequences in the nonredundant sequence database, and we reasoned that a conventional PSSM computed from sequence homologs might not be a sufficiently accurate guide for design calculations.

To address the limited diversity of PvRBP2b homologs in nature, we experimented with a different strategy for generating a PSSM using ProteinMPNN (38). ProteinMPPN uses a graph neural network architecture trained on structures and sequences observed in the Protein Data Bank (PDB) to design new sequences given a protein backbone without relying on information from homologs. This approach was successfully applied to *de novo* designs, the majority of which were based on small α-helical structures (34). Due to the high α-helix content of PvRBP2b_169-470_ (79%), we hypothesized that ProteinMPNN may make accurate predictions in this protein. However, previous applications of ProteinMPNN to natural proteins (which are typically larger and topologically more complex than *de novo* designed ones) showed lower success rates than PROSS and required significant experimental screening (39), which would be impractical in the case of a complex protein such as PvRBP2b. To address this potential problem, rather than designing PvRBP2b variants entirely by ProteinMPNN, we used ProteinMPNN to generate a PSSM as a replacement for the natural homolog-based PSSM in the PROSS workflow. Our implementation therefore combines ProteinMPNN with atomistic design calculations and eliminates the requirement of a deep MSA of homologs (Fig. 1). In all calculations, we excluded positions involved in antibody interfaces from the designs to maintain the parental antigenic profile. We designed six variants with the standard phylogenetic strategy (encoding 20–37 mutations from WT; designs WHT2476–WHT2481) and three with the ProteinMPNN-based PSSMs that exhibited fewer mutations (17–26; designs WHT2482, WHT2483, and WHT2484) (Fig. 1 and Table S1).

### Designs have improved yields and increased thermal stability

**Figure 2:**
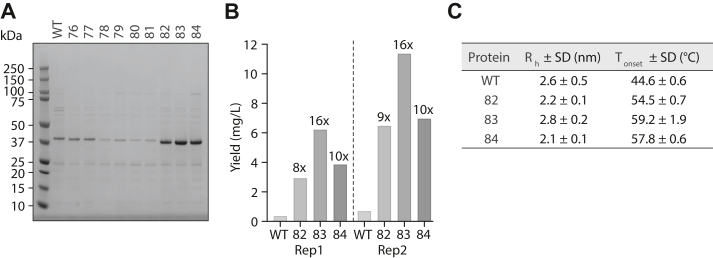
**Purification yields and biophysical characterization of parental PvRBP2b**_**169-470**_**and designs.***A*, Coomassie-stained SDS-PAGE gel of Ni-NTA affinity purified parental PvRBP2b_169-470_ and nine designs in reducing conditions. *B*, final yields (mg/L bacterial culture) for parental and stabilized designs after Ni-NTA affinity, ion exchange, and size-exclusion purification steps with fold change increase relative to parental yields shown on *top* of the corresponding bar graphs. The *dotted line* separates the two independent replicates for protein purification. *C*, dynamic light scattering (DLS) measurements of parental and three stabilized designs showing the hydrodynamic radius (*R*_h_) and unfolding onset temperatures *(T*_*onset*_) from two independent replicates. PvRBP2b, *Plasmodium vivax* reticulocyte-binding protein 2b.

We expressed parental PvRBP2b_169-470_ and the nine designs in *Escherichia coli* and used nickel-nitrilotriacetic acid affinity chromatography as the first purification step. SDS-PAGE analyses of purified proteins after nickel-nitrilotriacetic acid affinity chromatography showed that all three PROSS-MPNN PvRBP2b_169-470_ designs (WHT2482, WHT2483, and WHT2484) had increased yields relative to the parental construct (Fig. 2*A*), whereas only two designs (WHT2476 and WHT2477) from the standard PROSS workflow expressed as well as the parent. For the three PROSS-MPNN designs (WHT2482, WHT2483, and WHT2484), we proceeded with two additional purification steps using cation exchange and size-exclusion chromatography (Figs. 2*B* and S1, *A*–*C*). After the three-step purification process, WHT2482, WHT2483, and WHT2484 showed increased protein yields up to 9-, 16-, and 10-fold, respectively, compared to parental PvRBP2b_169-470_ (Fig. 2*B*). WHT2482, WHT2483, and WHT2484 had final yields up to 6, 11, and 7 mg/l of *E. coli* culture, respectively, compared to 0.7 mg per liter for the parental protein (Fig. 2*B*).

Dynamic light scattering measurements by regularization analysis showed that the parental protein and three designs were mainly monomeric with a similar hydrodynamic radius (Rh) in line with the theoretical molecular weight (Fig. 2*C*). However, the three designs displayed significantly higher thermal stability with *T*_*onset*_ values of 54 to 59 °C than 45 °C for the parental protein (Fig. 2*C*), translating to an improvement of 9 to 14 °C. We also conducted thermal stability measurements using a label-free differential scanning fluorimeter and observed that the designs showed higher thermal stability as described by the inflection temperature (T_i_) by 8 to 10 °C than parental PvRBP2b_169-470_ (Fig. S1*D*).

### Stabilized designs retain the PvRBP2b structural fold

**Figure 3:**
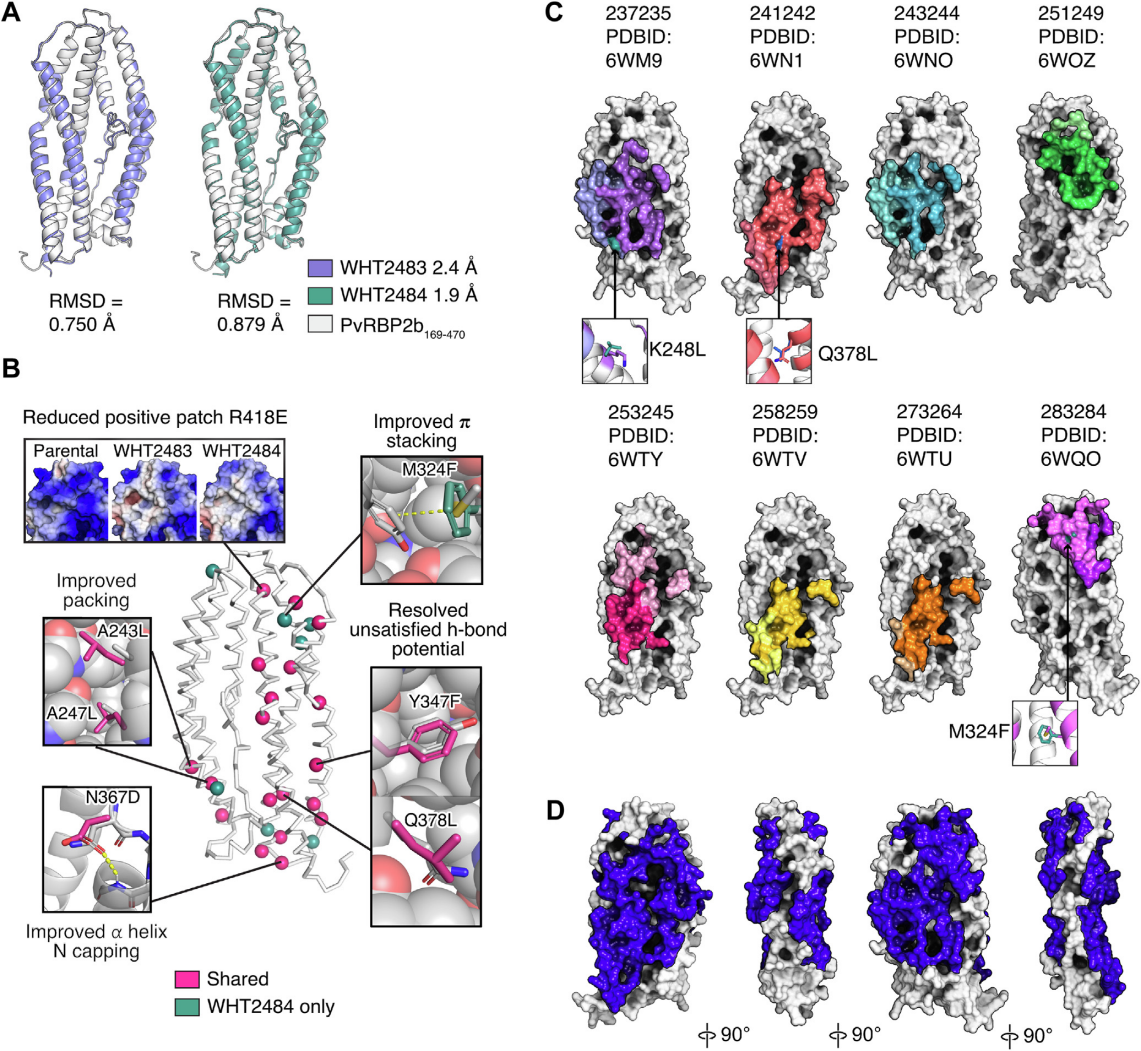
**Structural comparison of parental PvRBP2b**_**169-470**_**with stabilized designs WHT2483 and 2484.***A*, crystal structures of WHT2483 (*purple*) and WHT2484 (*green*) are overlayed with parental PvRBP2b_169-470_ with RMSD values provided. *B*, mutated residues are shown as *spheres* mapped onto *ribbon representations* of the parental PvRBP2b_169-470_ structure. Mutations present in both WHT2483 and WHT2484 are shown in *pink*, and mutations present only in WHT2484 are shown in *green*. Representative mutations that demonstrate stabilising effects are highlighted on the tertiary structure compared with parental PvRBP2b_169-470_. *C*, binding footprints of human antibodies against parental PvRBP2b_169-470_. Parental PvRBP2b_169-470_ is shown in *surface representation* (*white*), and *coloured regions* denote residues involved in antibody binding, with light chain interactions in a *lighter shade* and heavy chain interactions in a *darker shade* for each antibody. Interacting residues are obtained from previously published work (22) and were determined using PISA (55). Mutations within an antibody binding footprint are shown in *blue* for those in WHT2482 to 2484 inclusive (241242 Q378L) and *teal* for WHT2484 alone (237235 K248L and 283284 M324F). *D*, total antibody bound surface (*deep blue*) for the eight human mAbs is shown on parental PvRBP2b_169-470_ surface (*white*). PvRBP2b, *Plasmodium vivax* reticulocyte-binding protein 2b.

To determine whether the designs retained the structure of the parental protein, we determined the crystal structures of WHT2483 and WHT2484 (19 and 26 mutations, respectively) to 2.4 Å and 1.9 Å resolution, respectively (Fig. 3*A* and Table S2). We were unable to obtain any crystallization hits for WHT2482. An overlay of the structural coordinates of parental PvRBP2b_169-470_ (PDB ID 5W53) with WHT2483 and WHT2484 shows very similar structural scaffolds with RMSDs of 0.750 Å and 0.879 Å, respectively (9) (Fig. 3*A*). The biggest divergence in the backbones occurs at the C-terminal portions of the α5 and α7 helices with up to 1.7 Å difference in position at His464 in the α7 helix in WHT2484. However, this region does not interact with human antibodies against PvRBP2b (22).

To assess the reasons for the higher bacterial expression levels and improved thermal stability of the design, we compared their structure to that of the parental PvRBP2b_169–470_. This comparison showed several of the hallmarks expected for stabilizing mutations including a reduction in the size of homogenously charged surface patches, improved core packing, improved π stacking, and removal of buried polar residues that exhibit unsatisfied hydrogen bond donors or acceptors (Fig. 3*B* and Table S1). Most of these changes affect the solvent inaccessible interior of the protein, which are not directly involved in antibody recognition.

The main surface change is the reduction in positive charge in a patch on both WHT2483 and WHT2484 compared to the parental construct due to the introduction of the charge-swap mutation Arg418Glu (Fig. 3*B*). This is reflected in the overall reduction in the calculated pI from 9.2 for parent to 8.9 and 8.8 for WHT2483 and WHT2484, respectively. Apart from this region, however, the charge patterns remain similar (Fig. S2).

### PvRBP2b_169-470_ stabilized designs retain human mAb recognition

To ensure that the stabilized designs maintained the epitopes for human antibody recognition, we determined the binding kinetics and affinities of the interaction between human mAbs and WHT2482, WHT2483, and WHT2484 using biolayer interferometry (Table 1). These nine mAbs were chosen because they bind parental PvRBP2b_169–470_ with picomolar affinity, and we have high-resolution crystal structures of their antigen-binding fragments with this domain (22) (Fig. 3*C*). In addition, 48% of the antigen is engaged for interaction these human mAbs (Fig. 3*D*). Our results show that WHT2482, WHT2483, and WHT2484 bound all nine human antibodies with affinity in the low nanomolar range (Table 1 and Fig. S3). While the affinities were not uniformly as high compared to the parental PvRBP2b_169-470_, it was clear that the three stabilized designs retained recognition to a panel of naturally acquired mAbs.

**Table 1:** Human antibody affinities to parental PvRBP2b_169-470_ and three stabilized designs

HuMAb	Replicate 1					Replicate 2				
	WT					WT				
	K_D_ (nM)	K_a_ (× 10^5^ M^−1^s^−1^)	K_d_ (× 10^−5^ s^−1^)	X^2^	R^2^	K_D_ (nM)	K_a_ (× 10^5^ M^−1^s^−1^)	K_d_ (× 10^−5^ s^−1^)	X^2^	R^2^
237235	<0.001	17.83	<0.01	0.076	0.996	<0.001	13.06	<0.01	0.065	0.997
241242	<0.001	15.19	<0.01	0.137	0.994	<0.001	12.42	<0.01	0.092	0.995
243244	<0.001	17.49	<0.01	0.092	0.995	<0.001	13.24	<0.01	0.090	0.994
251249	<0.001	16.30	<0.01	0.030	0.998	<0.001	12.46	<0.01	0.022	0.998
253245	<0.001	15.84	<0.01	0.127	0.994	<0.001	11.77	<0.01	0.056	0.996
258259	<0.001	13.31	<0.01	0.089	0.996	<0.001	9.76	<0.01	0.045	0.998
260261	<0.001	13.09	<0.01	0.117	0.996	<0.001	12.03	<0.01	0.097	0.996
273264	<0.001	11.77	<0.01	0.148	0.996	<0.001	10.00	<0.01	0.041	0.998
283284	<0.001	14.81	<0.01	0.033	0.998	<0.001	9.94	<0.01	0.063	0.995
239229	NB	NB	NB	NB	NB	NB	NB	NB	NB	NB
250233	NB	NB	NB	NB	NB	NB	NB	NB	NB	NB


HuMAb	WHT2482					WHT2482				
	K_D_ (nM)	K_a_ (× 10^5^ M^−1^s^−1^)	K_d_ (× 10^−5^ s^−1^)	X^2^	R^2^	K_D_ (nM)	K_a_ (× 10^5^ M^−1^s^−1^)	K_d_ (× 10^−5^ s^−1^)	X^2^	R^2^
237235	<0.001	10.85	<0.01	0.024	0.999	0.028	13.59	3.83	0.033	0.998
241242	<0.001	10.54	<0.01	0.041	0.998	0.040	13.27	5.33	0.029	0.998
243244	<0.001	11.26	<0.01	0.010	0.999	<0.001	13.12	<0.01	0.022	0.999
251249	<0.001	11.31	<0.01	0.010	0.999	0.076	13.74	10.48	0.016	0.999
253245	<0.001	10.99	<0.01	0.027	0.998	0.010	14.05	1.41	0.018	0.999
258259	<0.001	7.38	<0.01	0.011	0.999	0.079	10.42	8.26	0.011	0.999
260261	0.190	6.91	13.15	0.028	0.996	<0.001	5.48	<0.01	0.011	0.999
273264	0.197	9.56	18.81	0.024	0.999	0.049	11.02	5.40	0.031	0.998
283284	<0.001	9.97	<0.01	0.015	0.999	<0.001	10.49	<0.01	0.011	0.999
239229	NB	NB	NB	NB	NB	NB	NB	NB	NB	NB
250233	NB	NB	NB	NB	NB	NB	NB	NB	NB	NB


HuMAb	WHT2483					WHT2483				
	K_D_ (nM)	K_a_ (× 10^5^ M^−1^s^−1^)	K_d_ (× 10^−5^ s^−1^)	X^2^	R^2^	K_D_ (nM)	K_a_ (× 10^5^ M^−1^s^−1^)	K_d_ (× 10^−5^ s^−1^)	X^2^	R^2^
237235	0.010	97.81	1.00	0.017	0.999	0.021	12.04	25.59	0.035	0.998
241242	<0.001	98.98	<0.01	0.012	0.999	0.026	12.58	33.02	0.044	0.997
243244	0.053	10.25	5.41	0.017	0.999	0.018	12.40	22.44	0.027	0.998
251249	<0.001	9.78	<0.01	0.013	0.999	0.029	13.73	39.84	0.044	0.997
253245	<0.001	10.43	<0.01	0.017	0.999	0.172	12.58	21.58	0.034	0.998
258259	0.085	7.78	6.59	0.028	0.999	0.059	9.49	5.60	0.013	0.999
260261	<0.001	5.40	<0.01	0.019	0.998	<0.001	5.03	<0.01	0.007	0.999
273264	0.089	7.78	6.92	0.020	0.999	0.154	10.71	16.47	0.020	0.999
283284	0.020	8.38	1.65	0.019	0.999	<0.001	9.10	<0.01	0.015	0.999
239229	NB	NB	NB	NB	NB	NB	NB	NB	NB	NB
250233	NB	NB	NB	NB	NB	NB	NB	NB	NB	NB


HuMAb	WHT2484					WHT2484				
	K_D_ (nM)	K_a_ (× 10^5^ M^−1^s^−1^)	K_d_ (× 10^−5^ s^−1^)	X^2^	R^2^	K_D_ (nM)	K_a_ (× 10^5^ M^−1^s^−1^)	K_d_ (× 10^−5^ s^−1^)	X^2^	R^2^
237235	<0.001	6.58	<0.01	0.016	0.998	0.339	7.45	25.27	0.031	0.997
241242	0.040	9.56	3.78	0.027	0.998	0.251	11.82	29.70	0.037	0.998
243244	0.109	7.05	7.66	0.022	0.997	0.353	7.80	27.48	0.026	0.997
251249	0.110	9.56	10.54	0.024	0.998	0.314	12.56	39.48	0.034	0.997
253245	0.001	10.58	0.11	0.032	0.998	0.034	11.19	3.81	0.024	0.998
258259	0.069	7.78	5.39	0.030	0.998	0.081	9.53	7.69	0.019	0.999
260261	0.053	8.90	4.74	0.026	0.996	0.209	5.49	11.47	0.034	0.994
273264	0.139	8.93	12.39	0.015	0.999	0.101	10.49	10.62	0.014	0.999
283284	0.112	10.08	11.32	0.021	0.998	0.074	9.36	6.89	0.020	0.998
239229	NB	NB	NB	NB	NB	NB	NB	NB	NB	NB
250233	NB	NB	NB	NB	NB	NB	NB	NB	NB	NB

Table containing determined kinetic and affinity data from two independent experiments including values for *K*_*D*_, *k*_*a*_, *k*_*d*_, *X*^*2*^, and *R*^*2*^ values as measured by bio-layer interferometry.

NB, nonbinding; PvRBP2b, *Plasmodium vivax* reticulocyte-binding protein 2b.

### Stabilized designs function as reliable serological markers for recent *P. Vivax* infection

**Figure 4:**
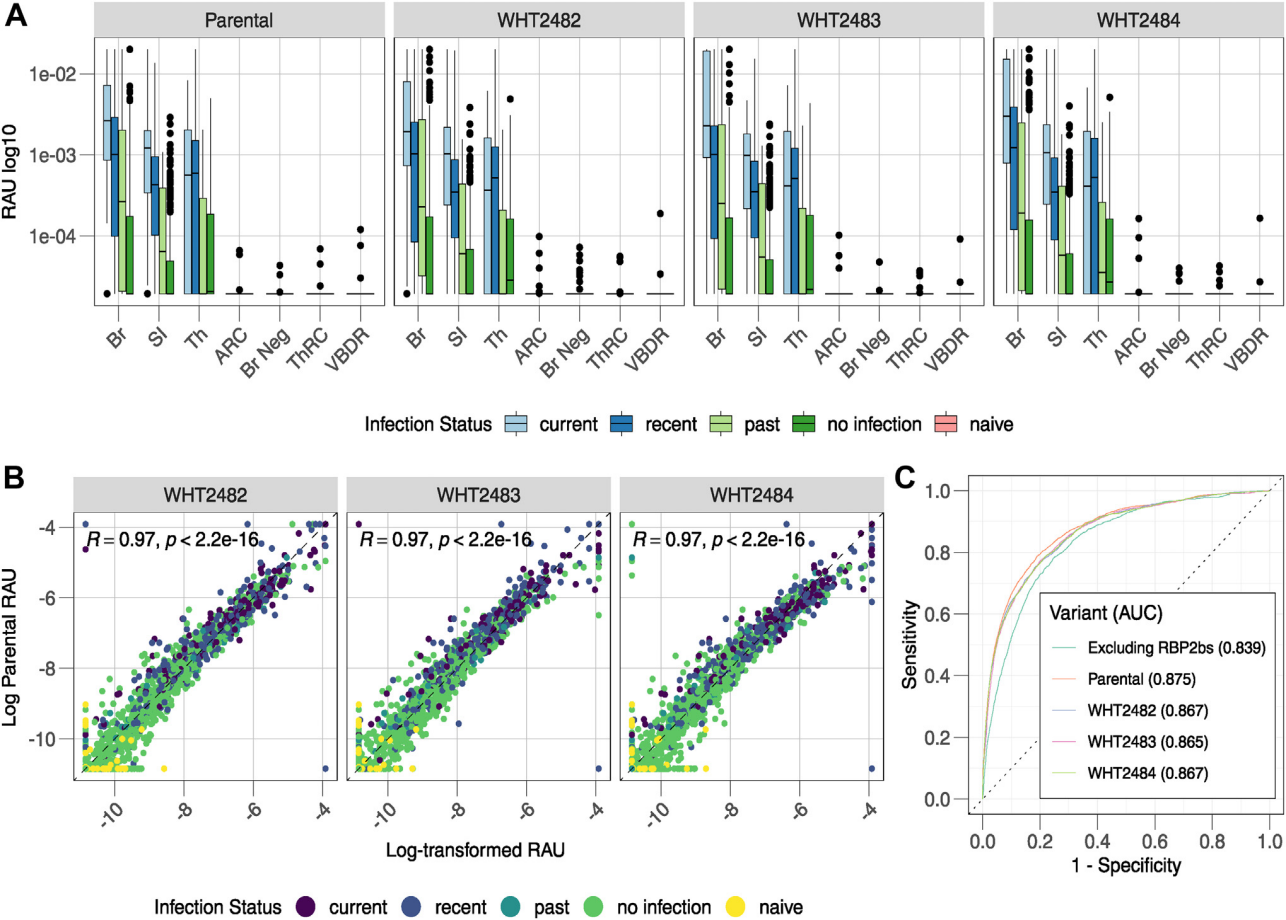
**Stabilized PvRBP2b**_**169-470**_**designs function as reliable serological markers for recent *P. vivax* infection.***A*, antibody responses toward PvRBP2b proteins measured in relative antibody units (RAUs) from the year-long cohort studies, as well as negative controls from Australian Red Cross (ARC), Brazil (Br Neg), Thai Red Cross (ThRC), and the Volunteer Blood Donor Registry (VBDR) in Victoria, Australia. Boxplots illustrate the median and 25th and 75th percentiles of the distribution of antibody responses for individuals who had a (i) current infection (*i.e.*, positive qPCR results for *P. vivax* at the time of antibody measurement), (ii) recent infection within the previous 9 months, (iii) old infection (*i.e.*, infection nine to 12 months ago), (iv) no infection during the year-long cohort study, and (v) the negative controls. *B*, correlation between parental PvRBP2b_169-470_ and designs and associated Pearson correlation coefficient (R), with colors representing the infection status as indicated. *C*, receiver operating characteristic (ROC) curve for eight-antigen sero-diagnostic combination comparing parental PvRBP2b and designs and the associated area under the curve (AUC). PvRBP2b, *Plasmodium vivax* reticulocyte-binding protein 2b.

To ensure that the stabilized designs maintain recognition of human polyclonal antibody responses, we used a multiplexed Luminex assay to measure IgG antibody responses against the parental construct and WHT2482, WHT2483, and WHT2484 in individuals from malaria-endemic regions of Brazil, Thailand, and the Solomon Islands (Fig. 4*A*). Like the IgG response against the parental construct, design-specific IgG responses were highest in individuals with current and recent (prior 9 months) *P. vivax* infections, declining with increasing time since the last detected *P. vivax* infection (Fig. 4*A*). Additionally, as per the parental construct, design-specific background IgG responses in malaria-naïve control samples from nonendemic areas were minimal. The variant-specific IgG responses were highly correlated with parental PvRBP2b_169-470_ (Pearson’s *r* = 0.97, *p* < 0.001) (Fig. 4*B*). To classify recent *P. vivax* exposure, we previously demonstrated that combinations of IgG responses against multiple *P. vivax* proteins provides better accuracy than using responses to single proteins (5). We therefore tested the ability of the designs to replace the parental construct in a random forest classification algorithm and measured the impact on performance. Comparable sensitivity, specificity, and area under the curve (AUC) values were obtained for the parent and the designs as demonstrated by the receiver operator characteristic curves (Fig. 4*C*, AUCs >0.865). An alternative option for diagnostic development is to replace PvRBP2b with a different *P. vivax* antigen which is more stable. However, we demonstrated substantial loss of performance when no PvRBP2b antigen was included (Fig. 4*C*, AUC 0.839), showing the importance of PvRBP2b in the diagnostic classification.

## Discussion

We have generated stabilized designs of the leading *P. vivax* sero-diagnostic candidate PvRBP2b that exhibit improved expression yields and thermal stability, while retaining antigenicity and diagnostic classification performance. This generated an improved recombinant antigen that may be more economical to produce and thermally stable for delivery and long-term storage. Cost of production and refrigeration-free transport are two critical determinants for the feasibility of diagnostics intended for use in developing countries (4), and our designs may aid efforts to implement PvRBP2b-based diagnostics in endemic regions.

PvRBP2b belongs to the PvRBP family of proteins which are homologous to the PfRh family (7, 8, 40). Several members of the PvRBP and PfRh family are known to be important in red blood cell invasion and their human red blood cell receptors have been identified; PvRBP2b binds to TfR1, PvRBP2a binds to CD98, PfRh5 binds to basigin and PfRh4 binds to Complement Receptor 1. The crystal structure of the N-terminal domain of PvRBP2b closely resembles the structures of the homologous domains of PvRBP2a and PfRh5 (9, 10, 41, 42, 43). While several PvRBP and PfRh proteins have been successfully made in different expression systems, there exist only a handful of high-resolution crystal structures of these important parasite adhesins as described above. Given that the design approach we developed and validated generates stable designs while minimally perturbing antigenic surfaces, it may be applied to AI-based model structures of these adhesins to enable crystallographic analysis including of complexes. Such structures are critical for developing diagnostics and vaccine immunogens.

Comparison of the binding footprints of the human antibody panel with the mutations introduced in WHT2483 and WHT2484 reveals that the binding surfaces are largely conserved, with few of the introduced mutations being within the antibody bound surface of parental PvRBP2b_169-470_ (Fig. 3*C*). There are three introduced mutations which fall within the antibody binding footprints, these are Lys248Leu and Met324Phe which are present in WHT2484, as well as Gln378Leu which is present in both WHT2483 and WHT2484 (Fig. 3*C*). These mutations fall within the binding footprints of human mAbs 237235, 283284, and 241242, respectively. Despite the presence of these mutations, these human mAbs still bound with nanomolar affinities to the stabilized designs (Table 1). Recognition may be retained for Lys248 and Met324 as the contacts are solely mediated by van der Waals interactions with the sidechains, which could either be retained by the residue swap or are long range (>5.0 Å). The interaction of 241242 with Gln378 is mediated through the peptide backbone which would remain unchanged in the stabilized designs.

This work builds upon our prior research demonstrating that PvRBP2b is currently the most important *P. vivax* antigen in the proposed serological biomarker panel (5) and clearly demonstrates the importance of our approach to improve thermal stability and yield, as exclusion of any PvRBP2b protein in the classification algorithm resulted in decreases in performance to a level that likely deteriorates the reliability of the serological test and treatment approach (44). While there may be approaches to overcome this drop in performance, such as repeated rounds of serological screening or better coverage (23, 44), these are likely to increase the overall cost of the public health interventions such as serological testing and treatment intervention and burden to participants and health workers.

The approach used here is also highly applicable for the development of other serological biomarkers which require increased stability while maintaining immunogenicity. Notably, the three PROSS-MPNN designs were all stabilized and highly expressed relative to the parental protein, demonstrating the high reliability of this design strategy. This approach therefore enables rapid and significant enhancement of stability and expression levels in one test cycle, rather than relying on laborious cycles of design and experimental testing which would be impractical for a 39 kDa protein with multiple different binding partners. We therefore expect this method to contribute to serological biomarker and vaccine production across a wide range of infectious diseases affecting humanity.

PROSS has been successfully applied to dozens of challenging proteins of biomedical and technological use (25). Nevertheless, its reliance on crystallographic structures and diverse sequence homologs has restricted its application. The advent of AI-based structure predictors, such as AlphaFold, has eliminated the need for crystallographic structures in many cases (26, 45). Now, our results show that AI-based sequence generators, such as ProteinMPNN, may further eliminate the need for diverse sequence homologs; we note, however, that the reliability of sequence generators in proteins that exhibit a low fraction of secondary-structure content should be carefully examined. Together, these advances in protein modeling and design may expand the reach of computational protein engineering, in principle, to any known protein.

## Experimental procedures

### PROSS stability design

PvRBP2b crystal structures were analyzed to identify regions that interact with human antibodies from hydrogen deuterium exchange mass spectrometry and crystallography data (22) (PBD ID: 5W53, 6WM9, 6WN1, 6WNO, 6WOZ, 6WTY, 6WTV, 6WTU, and 6WQO). Surfaces in these regions were disabled from design (Fig. 3, *C* and *D*). For designs WHT2476 to WHT2481, the PROSS protocol was applied as described in the original article (25). For designs WHT2482 to WHT2484, we replaced the MSA-based PSSM by a pseudo-PSSM derived from the statistical representation of parental PvRBP2b_169-470_ by ProteinMPNN (38). Using the backbone coordinates of parental PvRBP2b_169-470_ (PBD ID: 5W53) (9), ProteinMPNN built a backbone-specific mutation model. Log likelihoods for amino acid substitution at each position were extracted from the model. Probabilities were averaged over 50 independent initializations to account for stochastic variations and rounded to the closest integer. The resulting matrix was then treated as a standard PSSM within the PROSS workflow. Subsequent structure-based energy filtering of mutations and design steps were performed exactly as in the PROSS protocol for all designs. Final designs were analyzed visually and point mutations with low alpha helix propensity were eliminated prior to DNA synthesis.

### PvRBP2b_169–470_ and designs cloning and sequencing

Sequence of PvRBP2b from *P. vivax* strain Salvador I was obtained from PlasmoDB Database (www.plasmodb.org; accession number: PVX_094255, 2806 amino acids). Synthetic DNA was codon-optimized for expression in *E. coli* (Life Technologies). The nucleotide sequence encoding amino acids 169 to 470 of PvRBP2b was cloned into pPROEX HTb vector which included sequences for a N-terminal 6xHis-tag followed by a TEV cleavage site (9). This sequence refers to the parental PvRBP2b_169-470_. Restriction enzyme cloning was used to clone the synthetic DNA fragments (obtained from Twist Biosciences) of PvRBP2b_169-470_ designs into pPROEX HTb vector. All positive plasmids were sequence verified at the WEHI Advanced Genomics Facility.

### Expression and purification of PvRBP2b_169–470_ designs

Parental PvRBP2b_169–470_ was expressed using *E. coli* strain SHuffle T7 (New England BioLabs) and terrific broth supplemented with 100 μg/ml of carbenicillin (9). Flasks containing 1 L of medium were incubated in a Multitron shaker (Infors HT) at 37 °C at 180 rpm. At OD_600_ of around 1.0, IPTG (Astral) was added to the final concentration of 1.0 mM and protein expression was allowed to continue for 20 h at 16 °C. Cells were harvested by centrifugation at 6000*g*, resuspended in freezing buffer containing 50 mM Tris–HCl pH 7.5, 500 mM NaCl, 10% (v/v) glycerol supplemented with cOmplete EDTA-free protease inhibitor cocktail (Roche), and stored at −80 °C until further processing.

For the purification, cell pellet was thawed on ice and resuspended in the freezing buffer supplemented with 0.5 mg/ml of DNase and 1.0 mg/ml of lysozyme (Sigma-Aldrich). Cells were lysed using sonicator Sonopuls UW 3200 (Bandelin) equipped with VS 70 T probe. The obtained crude cell extract was clarified by centrifugation at 30,000*g* for 45 min at 4 °C. The supernatant was loaded onto the 5 ml HisTrap excel column (GE Healthcare) pre-equilibrated with the freezing buffer. Unbound material was removed using 10 column volumes of wash buffer: 20 mM Tris–HCl pH 7.5, 500 mM NaCl, and 10 mM imidazole. The bound protein was eluted from the column using the same buffer but containing 300 mM imidazole. Eluted fractions were pooled and dialyzed overnight into the dialysis buffer containing 20 mM Tris–HCl pH 7.5 and 100 mM NaCl. The resulting protein sample was applied on the 5 ml HiTrap SP HP cation exchange chromatography column (GE Healthcare) pre-equilibrated with the dialysis buffer. Unbound material was removed using 10 column volumes of the buffer. Protein was eluted from the column using a gradient of 20 mM Tris–HCl pH 7.5 and 1.0 M NaCl. Collected fractions were analyzed on SDS-PAGE and fractions of interest were concentrated using an Amicon Ultra-4 10 kDa molecular weight cut-off concentrator (Millipore) and loaded onto S75 Superdex 16/600 size-exclusion column (GE Healthcare) pre-equilibrated with 20 mM Hepes pH 7.5 and 150 mM NaCl. The monodisperse peak fractions containing protein were pooled and concentrated using the same concentrator, flash-frozen in liquid nitrogen, and stored at −80 °C. Expression and purification of WHT2482, WHT2483, and WHT2484 designs were performed in a similar manner as described above.

### Thermal stability assays

Parental PvRBP2b_169–470_ and designs were diluted to 1 mg/ml and transferred to an Aurora 384-well plate for dynamic light scattering measurements in the DynaPro plate reader III (Wyatt Technology). All samples were filtered, then centrifuged at 17,000*g* for 5 min at 4 °C with transfer to the plate wells. The plate was sealed, and measurements were performed over a temperature ramp of 25 to 80 °C at a ramp rate of 0.1 °C/min, with 4 s acquisition time averaged over 5 acquisitions. The onset model was fit to the data to obtain *T*_*onset*_ values for all constructs. *T*_*onset*_ values are the temperature at which the protein begins to unfold. Data from at least two different batches of protein in triplicate were evaluated using Dynamics software v8.0.0.89.

Thermal shift assays were performed using the Tycho NT.6 (NanoTemper Technologies). Parental PvRBP2b_169–470_ and designs were measured at 10 μM in storage buffer. Ten microliters of each sample was transferred into a capillary and measured from 35 to 95 °C using a Tycho NT.6 (Nanotemper). The inflection temperatures of each protein were calculated by the Tycho NT.6 software (1.2.0.750). Technical triplicates were measured in three independent experiments. Data were analyzed using GraphPad Prism (https://www.graphpad.com/).

### Crystallography methods

Crystallization trials were undertaken at the Bio21 Collaborative Crystallization Facility at 20 °C using 96-well sitting drop vapour diffusion plates (Greiner). Crystals were obtained for WHT2483 from a solution containing 5% (v/v) 2-Methyl-2,4-pentanediol, 10% (w/v) PEG 6000, and 0.1 M Hepes pH 7.5 at 10 mg/ml. Crystals were obtained for WHT2484 using 10% (v/v) propan-2-ol, 10% (w/v) PEG MME 5000, and 0.1 M Na cacodylate pH 6 at 15 mg/ml. Crystals were flash-frozen in liquid nitrogen at 100 K following cryoprotection with 15 to 20% glycerol in reservoir solution. Datasets were collected to 2.4 Å (WHT2483) and 1.9 Å (WHT2484) using the MX2 beamline at the Australian Synchrotron. Data were recorded using an Eiger 16M detector (Dectris) and processed using the XDS package (https://xds.mr.mpg.de/) (46). Molecular replacement was undertaken using Phaser (47). A search model was generated using AlphaFold2 models for WHT2483 and WHT2484, respectively (48).

The WHT2483 structure was solved in space group P2_1_2_1_2_1_ with two copies in the asymmetric unit, while WHT2484 was solved in space group P4_1_2_1_2 and had a single copy in the asymmetric unit. Refinement through iterative rounds of model building in COOT (https://www2.mrc-lmb.cam.ac.uk/personal/pemsley/coot/) (49) and refinement in Phenix v1.19.2 (https://phenix-online.org/) generated models with an R_obs_/R_free_ = 19.52/24.53 for WHT2483 and 18.88/22.17 for WHT2484 (50). Structural models were deposited in the PDB under PDB ID 9DZC and 9DZD, respectively. Figures were prepared using the PyMOL Molecular Graphics System (The PyMOL Molecular Graphics System, Version 3.0 Schrödinger, LLC; https://www.pymol.org/).

### Antibody expression and purification

Recombinant human mAbs were expressed in Expi293 HEK cells (Life Technologies) maintained in suspension at 37 °C and 8% CO_2_ (22). Cells were transfected at a density of 3 × 10^6^ with equal amounts of heavy and light-chain paired plasmids usingPEI (Sigma-Aldrich) at a ratio of 1:3 of the total amount of plasmid to PEI. One day after transfection, valproic acid was added to cultures to a final concentration of 0.025 M. Seven days after transfection, the supernatant was collected by centrifugation, and filtered through a 0.22 μm filter. Human mAbs were purified by loading the supernatant onto a 1 ml protein A HP HiTrap column (GE Healthcare). Columns were equilibrated and washed using Dulbecco’s PBS. Human mAbs were eluted using 0.1 mM citric acid pH 3.00 and neutralized with 1 M Tris–HCl pH 9.0. A second purification step was performed by loading protein A eluate on a Hiload 16/600 Superdex 200 pg gel filtration column (GE Healthcare), which was pre-equilibrated with Dulbecco’s PBS. Human mAbs were concentrated using Amicon Ultra-4 50 kDa (Millipore). Antibody concentration was determined by absorbance measurement at 280 nm using a Nanodrop spectrophotometer and purity was determined using SDS-PAGE.

### Biolayer interferometry

Antibody affinities were measured using an Octet RED96 instrument. Assays were performed at 25 °C in solid black 96-well plates agitated at 1000 rpm. The kinetic buffer was composed of PBS 0.1% bovine serum albumin, 0.05% Tween-20. A 60 s biosensor baseline step was applied before human mAbs were loaded onto anti-human IgG Fc capture sensor tips by submerging sensor tips in 5 μg/ml human mAb until a response of 0.5 nm then washed in a kinetic buffer for 60 s. Association measurements were performed using a 2-fold concentration gradient of parental PvRBP2b_169–470_ and designs from 0.63 to 10 nM for 200 s and dissociation was measured in a kinetic buffer for 300 s. Sensor tips were regenerated using a cycle of 5 s in 100 mM glycine pH 1.5 and 5 s in kinetic buffer repeated five times. Baseline drift was corrected by subtracting the average shift of a human mAb loaded sensor not incubated with parental PvRBP2b_169–470_ and designs and an unloaded sensor incubated with parental PvRBP2b_169–470_ and designs. Curve fitting analysis was performed with Octet Data Analysis 10.0 software using a global fit 1:1 model to determine *K*_D_ values and kinetic parameters. Curves that could not be reliably fitted were excluded from further analysis.

### Multiplex assay

A multiplexed Luminex magnetic bead–based assay was utilized as previously described (51). Briefly, parental PvRBP2b_169–470,_ designs WHT2482, WHT2483, and WHT2484, and additional *P. vivax* antigens utilised in combination algorithms, were coupled to individual sets of internally labeled magnetic COOH beads, following bead activation using 50 mg/ml sulfo-NHS and 50 mg/ml EDC. Details on additional proteins and the amount coupled is provided in Table S3. Antigen-specific total IgG antibodies were then detected in plasma samples by incubating 50 μl coupled beads with 50 μl 1/100 dilution of human plasma, followed by addition of 1/100 detector antibody (PE-conjugated anti-human IgG). On each plate, a 2-fold serial dilution from 1/50 to 1/25,600 of a positive control plasma pool (generated from adults from PNG) was included to enable conversion of the raw mean fluorescent intensity to an arbitrary relative antibody unit as previously described (51). Plates were read on a MAGPIX instrument as per the manufacturer’s instruction, with data acquired from at least 15 beads per region.

The described multiplexed assay was used to measure antigen-specific IgG antibodies in plasma samples from malaria-endemic regions in Thailand, Brazil, and the Solomon Islands as previously described (5). Briefly, yearlong cohort studies were conducted in Thailand (Kanchanaburi and Ratchaburi provinces), Brazil (Manaus), and Solomon Islands across 2013 to 2014 (52, 53, 54). Each site enrolled between 999 and 1274 individuals with blood samples taken every month for quantitative polymerase chain reaction detection of blood-stage *P. vivax* infections and plasma stored for antibody measurements. In the current study, we measured total IgG antibody responses at the final visit of the yearlong cohorts, enabling the magnitude to be related to the time since prior *P. vivax* infection.

### Statistical approaches and classification algorithm

We trained and tested random forest classification algorithms using the antibody responses toward the panel of *P. vivax* antigens as predictor variables and infection status as the outcome variable. Individuals infected within the previous 9 months were classified as recently infected and those with a *P. vivax* infection prior to 9 months, or no prior infection were classified as not recently infected. We compared the performance of a random forest classification algorithm trained on a panel of *P. vivax* antigens, while swapping the parental PvRBP2b_169-470_ for designs WHT2482, WHT2483, and WHT2484 within this multiantigen combination. We assess the classification performance using the area under the receiver operating curve (AUC). Details of the random forest are outlined in the supplementary information.

## Data availability

Structural models were deposited in the PDB under PDB ID 9DZC and 9DZD.

## Supporting information

This article contains supporting information.

## Conflict of interest

S. J. F. is a named inventor on patent filings related to design methods mentioned in the manuscript and consults on protein design. I. M. and R. J. L. are named inventors on a patent related to *P. vivax* serological exposure markers (PCT/US17/67926). Some of the data described is subject to provisional patent protection (AU2024904177). The authors declare that they have no conflicts of interest with the contents of this article.
