# Supplementary material for: Stabilized designs of the malaria adhesin protein PvRBP2b for use as a potential diagnostic for Plasmodium vivax[image]
Source: J Biol Chem. 2025 Feb 10;301(3):108290. doi: 10.1016/j.jbc.2025.108290 (PMC11929097; doi:10.1016/j.jbc.2025.108290)
Supplement: Figure S1 [file mmc1.pdf]

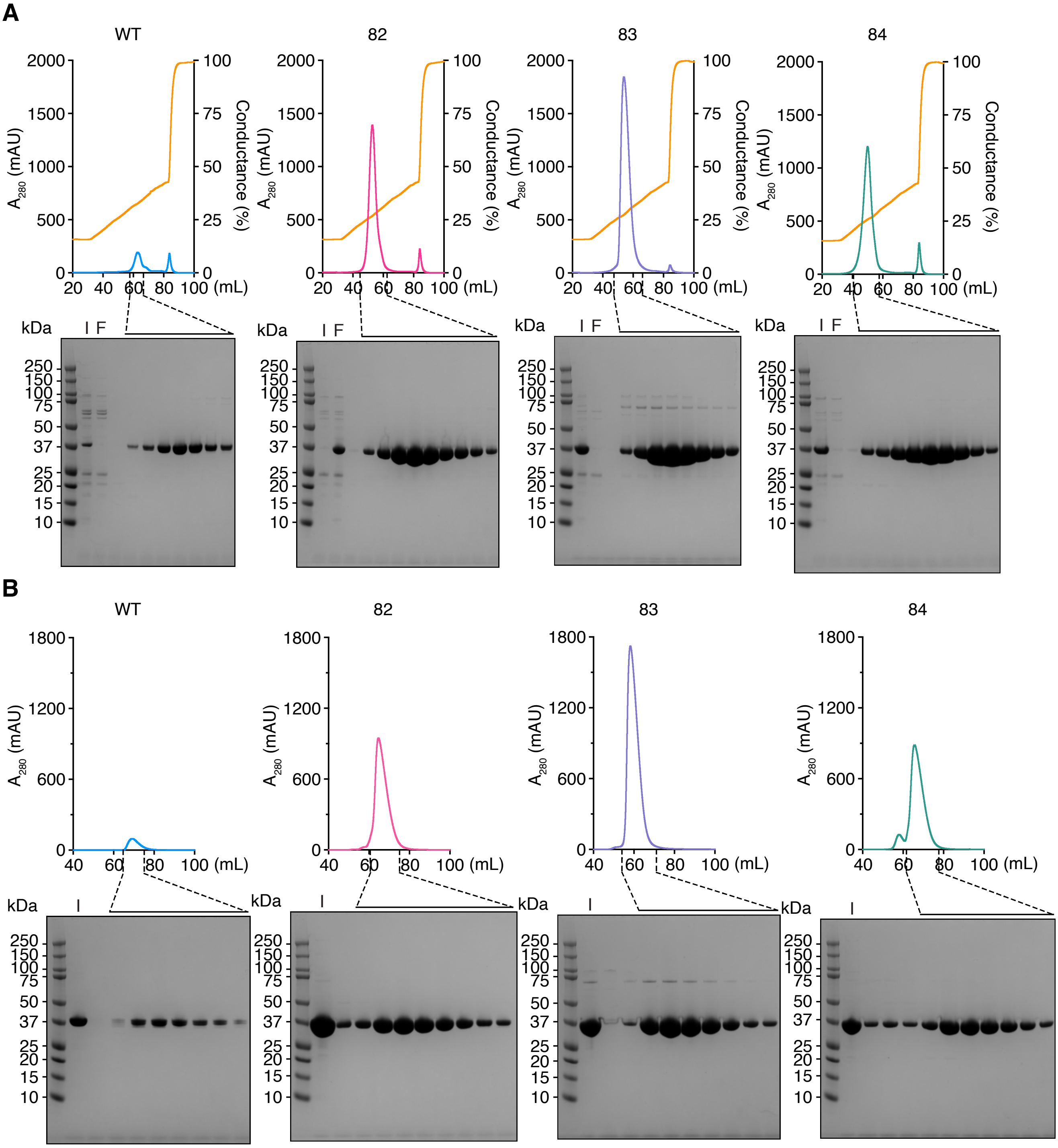

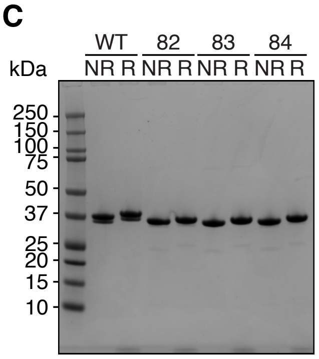

D

Protein	T_i1 ± SD (°C)	T_i2 ± SD (°C)
WT	63.7 ± 0.3	–
82	72.4 ± 0.4	–
83	74.1 ± 0.2	84.3 ± 0.5
84	73.1 ± 0.6	–

Figure S1. Purification and label-free differential scanning fluorimetry of parental PvRBP2b$_{169\text{-}470}$ and three stabilized designs. (A) Ion exchange chromatography (IEX) chromatograms and corresponding reduced SDS-PAGE gels of parental PvRBP2b$_{169\text{-}470}$ and designs. (B) Size exclusion chromatography (SEC) chromatograms and corresponding reduced SDS-PAGE gels of parental PvRBP2b$_{169\text{-}470}$ and designs. (C) Non-reduced (NR) and reduced (R) SDS-PAGE gel of recombinant parental PvRBP2b$_{169\text{-}470}$ and designs after affinity, IEX and SEC purification steps. (D) Inflection temperatures (T_i) of parental PvRBP2b$_{169\text{-}470}$ and designs using label free thermal shift analysis (Tycho NT.6, Nanotemper).
