# Supplementary material for: Stabilized designs of the malaria adhesin protein PvRBP2b for use as a potential diagnostic for Plasmodium vivax[image]
Source: J Biol Chem. 2025 Feb 10;301(3):108290. doi: 10.1016/j.jbc.2025.108290 (PMC11929097; doi:10.1016/j.jbc.2025.108290)
Supplement: Figure S2 [file mmc2.pdf]

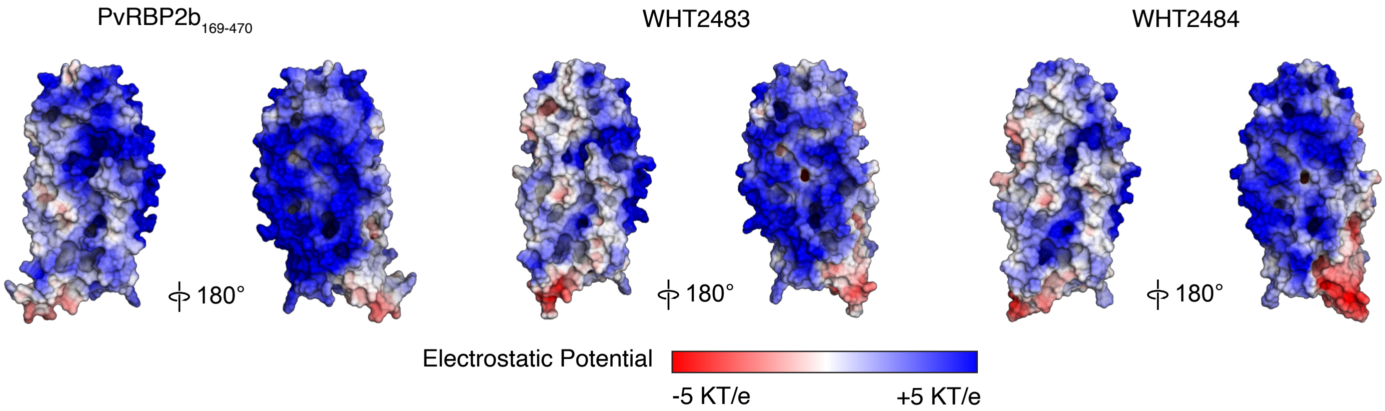

Figure S2. Electrostatic surfaces of PvRBP2b$_{169\text{-}470}$ and stabilized designs. Surface electrostatics calculations performed by the Adaptive Poisson-Boltzmann Solver (APBS) are displayed as blue (positive charge) and red (negative charge) formatting with a +/- 5 KT/e range on 180° rotated surface representations of parental PvRBP2b$_{169\text{-}470}$, WHT2483, and WHT2484.
