# Supplementary material for: Stabilized designs of the malaria adhesin protein PvRBP2b for use as a potential diagnostic for Plasmodium vivax[image]
Source: J Biol Chem. 2025 Feb 10;301(3):108290. doi: 10.1016/j.jbc.2025.108290 (PMC11929097; doi:10.1016/j.jbc.2025.108290)
Supplement: Figure S3 [file mmc3.pdf]

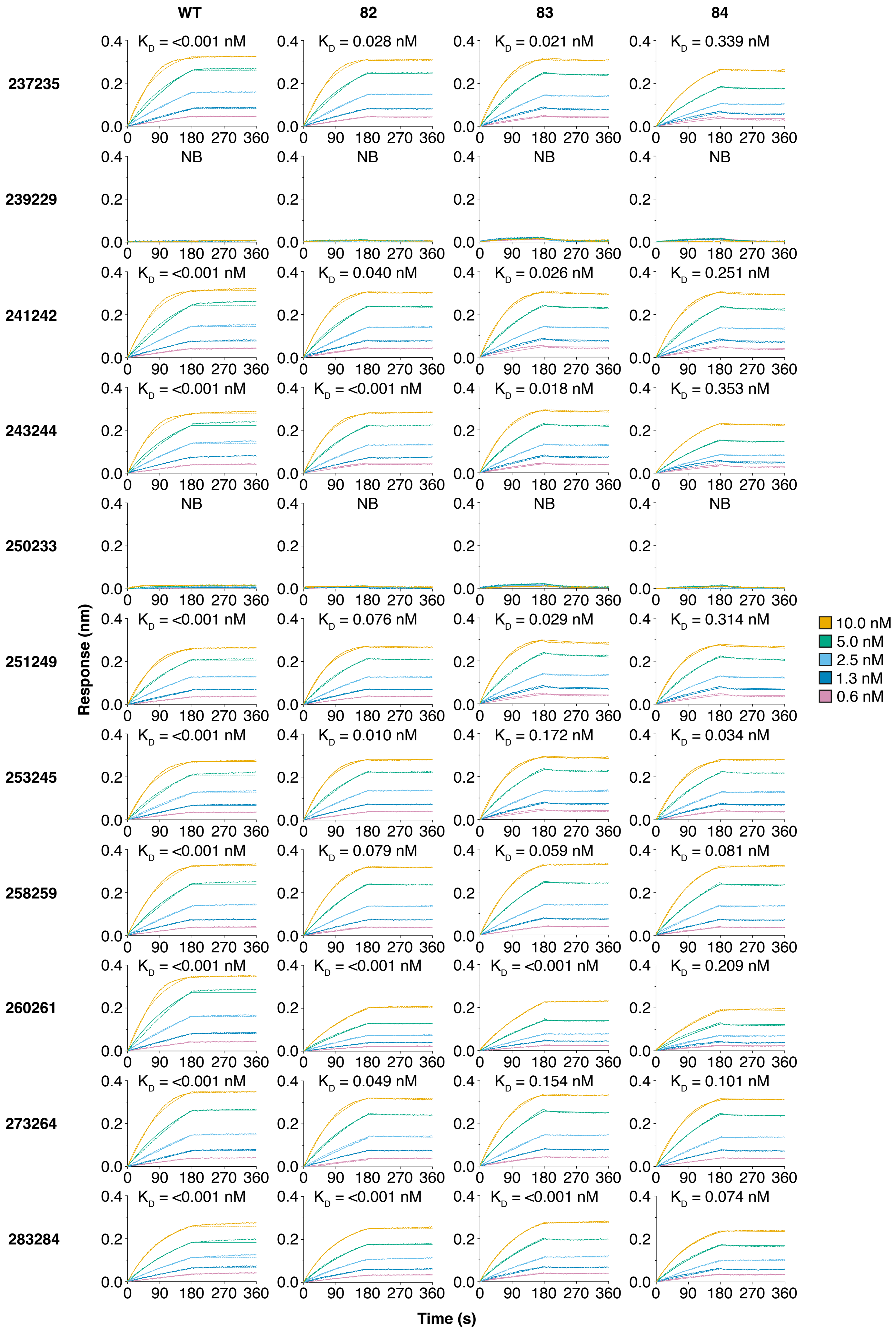

Figure S3. Representative BLI binding curves with PvRBP2b$_{169-470}$ designs and human monoclonal antibodies. Binding experiments were performed with five different concentrations from 0.6 - 10 nM of PvRBP2b$_{169-470}$ and stabilized designs. The measured binding curves are plotted (solid line) and fitted to a 1:1 binding model (dashed line). Representative binding curves are shown from two independent experiments. Corresponding K_D values are indicated.
