# Supplementary material for: Stabilized designs of the malaria adhesin protein PvRBP2b for use as a potential diagnostic for Plasmodium vivax[image]
Source: J Biol Chem. 2025 Feb 10;301(3):108290. doi: 10.1016/j.jbc.2025.108290 (PMC11929097; doi:10.1016/j.jbc.2025.108290)
Supplement: Table S1 [file mmc4.pdf]

Table S1. Mutated residues between parental $PvRBP2b_{169-470}$ and designs.

	WHT2476	WHT2477	WHT2478	WHT2479	WHT2480	WHT2481	WHT2482	WHT2483	WHT2484
1	R264N	R264N	R264N	R264N	R264N	R264N	A243L	A243L	A243L
2	V269E	V269E	V269E	V269E	V269E	V269E	V247L	V247L	V247L
3	E276T	E276T	E276T	E276T	E276T	E276T	K270L	K270L	K248L
4	K278L	K278L	K278L	K278L	K278L	K278L	F322W	F322W	K270L
5	G283N	G283N	A280E	A280E	A280E	A280E	Y337L	Y337L	K309R
6	D305K	D305K	G283N	G283N	G283N	G283N	Y347F	Y347F	F322W
7	Q317S	Q317S	K291E	K291E	K291E	K291E	G354Y	G354Y	M324F
8	N319K	N319K	D305K	D305K	D305K	D305K	M357L	M357L	Y337L
9	L352K	L352N	K309R	K309R	K309R	K309R	I361Y	N367D	Y347F
10	M357L	G354F	S313T	S313T	S313T	S313T	N367D	V369I	G354Y
11	G382K	D356N	Q317S	Q317S	Q317S	Q317S	T377V	I371L	M357L
12	S398A	M357L	N319K	N319K	N339D	N339D	Q378L	T377V	I361Y
13	D407N	V369D	N339D	N339D	Y347F	Y347F	S398A	Q378L	N367D
14	V409E	G382K	Y347F	Y347F	L352N	G354F	S406A	S398A	V369I
15	Q413Y	Q393E	L352N	G354F	G354F	M357L	R418E	S406A	I371L
16	N428Y	S398A	G354F	M357L	D356N	N367D	S431A	R418E	N372Q
17	S431A	V402I	D356N	N367D	M357L	V369D	G436A	S431A	T377V
18	G436S	D407N	M357L	V369D	N367D	S398A		G436A	Q378L
19	N444R	V409E	N367D	S398A	V369D	V402I		S439Q	S398A
20	E467K	Q413Y	V369D	V402I	G382K	A404D			V402I
21		N428Y	G382K	A404D	Q393R	D407N			L405I
22		S431A	Q393R	D407N	S398A	V409E			S406A
23		G436S	S398A	V409E	V402I	N417D			R418E
24		S439N	V402I	N417D	A404D	R418T			S431A
25		N444E	A404D	R418T	D407N	N428Y			G436A
26		G463S	D407N	N428Y	V409E	S431A			S439Q
27		E467K	V409E	S431A	N417D	G436S			
28			N417D	G436S	R418T	S439N			
29			R418T	S439N	N428Y	N444R			
30			N428Y	N444R	S431A	G459S			
31			S431A	G459S	G436S	G463S			
32			G436S	G463S	S439N	E467K			
33			S439N	E467K	N444R				
34			N444R		G459S				
35			G459S		G463S				
36			G463S		E467K				
37			E467K						
