# Supplementary material for: Stabilized designs of the malaria adhesin protein PvRBP2b for use as a potential diagnostic for Plasmodium vivax[image]
Source: J Biol Chem. 2025 Feb 10;301(3):108290. doi: 10.1016/j.jbc.2025.108290 (PMC11929097; doi:10.1016/j.jbc.2025.108290)
Supplement: Table S2 [file mmc5.pdf]

Table S2. Data collection and refinement statistics.

	WHT2483	WHT2484
**PDB ID**	9DZC	9DZD
**Data Collection**		
Resolution range	46.41 - 2.398 (2.46 - 2.4)	46.04 - 1.85 (1.9 - 1.85)
**Space group**	P 21 21 21	P 41 21 2
**Cell dimensions**		
a, b, c (Å)	79.237 92.828 118.205	46.423 46.423 361.005
α, β, γ (°)	90 90 90	90 90 90
R-merge	0.119 (1.066)	0.076 (1.463)
R-meas	0.128 (1.149)	0.082 (1.576)
R-pim	0.048 (0.425)	0.031 (0.582)
CC1/2	0.999 (0.806)	1.000 (0.792)
**Multiplicity**	13.5 (13.6)	12.7 (13.4)
**Completeness (%)**	99.8 (98.0)	100.0 (100.0)
**Mean I/sigma(I)**	15.7 (2.5)	19.5 (2.2)
**Wilson B-factor**	43.49	30.72
**Refinement**		
Total reflections	471555 (48302)	450878 (28118)
Unique reflections	34857 (3560)	35632 (2099)
R_{work} / R_{free} (%)	19.52 / 24.53	18.88 / 22.17
**Number of non-hydrogen atoms**	5348	2883
macromolecules	5109	2551
ligands	62	31
solvent	177	301
Protein residues	603	301
**RMS(bonds)**	0.002	0.008
RMS(angles)	0.54	0.83
Ramachandran		
Favored (%)	97.66	98.32
Allowed (%)	2.34	1.68
Outliers (%)	0	0
Rotamer outliers (%)	0.35	0.36
Clashscore	1.92	3.29
Average B-factor	52.58	36.36
macromolecules	52.30	35.08
ligands	78.21	63.96
solvent	51.77	44.35

X-ray diffraction data were collected on single crystals. Values for the highest-resolution shell are shown in parentheses.
