# Supplementary material for: Stabilized designs of the malaria adhesin protein PvRBP2b for use as a potential diagnostic for Plasmodium vivax[image]
Source: J Biol Chem. 2025 Feb 10;301(3):108290. doi: 10.1016/j.jbc.2025.108290 (PMC11929097; doi:10.1016/j.jbc.2025.108290)
Supplement: Table S3 [file mmc6.pdf]

Table S3. *P. vivax* proteins utilised in Luminex assays and the amounts coupled.

Protein	Gene ID (PlasmoDb)	Protein provider	Sequence region (amino acids)	Expression system	Amount (μg) coupled to 2.5 million Magplex beads
Parental PvRBP2b	PVX_094255	Wai-Hong Tham	169-470	*E. coli*	1.4
WHT2482	PVX_094255	Wai-Hong Tham	169-470	*E. coli*	1.4
WHT2483	PVX_094255	Wai-Hong Tham	169-470	*E. coli*	1.4
WHT2484	PVX_094255	Wai-Hong Tham	169-470	*E. coli*	1.4
EBP	KMZ83376.1	WEHI Protein Production Facility	1-716	Insect cells	0.93
PTEX150	PVX_084720	WEHI Protein Production Facility	23-908	Insect cells	1
Pv-fam-a	PVX_096995	ZiP Diagnostics	56-480	*E. coli*	3
MSP5	PVX_003770	ZiP Diagnostics	25-364	*E. coli*	0.3
MSP1-19	PVX_099980	ZiP Diagnostics	1623-1715	*E. coli*	4
MSP8	PVX_097625	WEHI Protein Production Facility	24-465	Insect cells	4
PvCSS	PVX_086200	WEHI Protein Production Facility	22-381	Mammalian	4
s16	PVX_000930	GenScript	30-92	chemical synthesis	100
RBP2a	PVX_121920	Wai-Hong Tham	160-1135	*E. coli*	10
