# Supplementary material for: Stabilized designs of the malaria adhesin protein PvRBP2b for use as a potential diagnostic for Plasmodium vivax[image]
Source: J Biol Chem. 2025 Feb 10;301(3):108290. doi: 10.1016/j.jbc.2025.108290 (PMC11929097; doi:10.1016/j.jbc.2025.108290)
Supplement: Table S4 [file mmc7.pdf]

Table S4. Top performing combination of antigens in random forest classification algorithm when using parental PvRBP2b compared to stabilized designs.

	Combination	AUC
PvRBP2b-parental	EBP + s16 + Pv-fam-a + MSP5 + Parental PvRBP2b + MSP1-19 + RBP2a + MSP8	0.875
WHT2482	EBP + PTEX150 + s16 + Pv-fam-a + MSP5 + WHT2482 + MSP1-19 + RBP2a	0.867
WHT2483	EBP + s16 + Pv-fam-a + MSP5 + WHT2483 + MSP1-19+ RBP2a + MSP8	0.865
WHT2484	EBP + PTEX150 + s16 + Pv-fam-a + MSP5 + WHT2484 + MSP1-19 + RBP2a	0.867
Excluding RBP2bs	MSP1-19 + Pv-fam-a + MSP5 + EBP + PTEX150 + PvCSS + s16 + RBP2a	0.839
